# Clinico-imaging features of subjects at risk of Lewy body disease in NaT-PROBE baseline analysis

**DOI:** 10.1038/s41531-023-00507-y

**Published:** 2023-04-26

**Authors:** Makoto Hattori, Keita Hiraga, Yuki Satake, Takashi Tsuboi, Daigo Tamakoshi, Maki Sato, Katsunori Yokoi, Keisuke Suzuki, Yutaka Arahata, Akihiro Hori, Motoshi Kawashima, Hideaki Shimizu, Hiroshi Matsuda, Katsuhiko Kato, Yukihiko Washimi, Masahisa Katsuno

**Affiliations:** 1 Department of Neurology, Nagoya University Graduate School of Medicine, Nagoya, Japan; 2 Department of Neurology and Center for Comprehensive Care and Research Center on Memory Disorders, National Center for Geriatrics and Gerontology, Obu, Aichi Japan; 3 Innovation Center for Translational Research, National Center for Geriatrics and Gerontology, Obu, Aichi Japan; 4 Kumiai Kosei Hospital, Takayama, Gifu Japan; 5 Medical Examination Center, Daido Clinic, Nagoya, Japan; 6 Department of Biofunctional Imaging, Fukushima Medical University, Fukushima, Japan; 7 Functional Medical Imaging, Biomedical Imaging Sciences, Division of Advanced Information Health Sciences, Department of Integrated Health Sciences, Nagoya University Graduate School of Medicine, Nagoya, Japan; 8 Department of Clinical Research Education, Nagoya University Graduate School of Medicine, Nagoya, Japan

**Keywords:** Parkinson's disease, Risk factors

## Abstract

Individuals with prodromal symptoms of Lewy body disease (LBD), such as rapid eye movement sleep behavior disorder (RBD), often showed imaging defects similar to patients with Parkinson’s disease and dementia with Lewy bodies. We examined dopamine transporter (DaT) single-photon-emission computed tomography (SPECT) and metaiodobenzylguanidine (MIBG) scintigraphy in 69 high-risk subjects with ≥2 prodromal symptoms (dysautonomia, hyposmia, and probable RBD) and 32 low-risk subjects without prodromal symptoms, whom were identified through a questionnaire survey of health checkup examinees. The high-risk subjects had significantly worse scores on Stroop test, line orientation test, and the Odor Stick Identification Test for Japanese than the low-risk subjects. The prevalence of abnormalities on DaT-SPECT was higher in the high-risk group than in the low-risk group (24.6% vs. 6.3%, *p* = 0.030). A decreased uptake on DaT-SPECT was associated with motor impairment, and MIBG scintigraphy defects were associated with hyposmia. The simultaneous evaluation of DaT-SPECT and MIBG scintigraphy may capture a wide range of individuals with prodromal LBD.

## Introduction

Lewy body disease (LBD) is a neurodegenerative disorder associated with the intra-neuronal accumulation of alpha-synuclein and includes Parkinson’s disease (PD) and dementia with Lewy bodies (DLB). PD and DLB share common prodromal symptoms, such as autonomic dysfunction, hyposmia, and rapid eye movement sleep behavior disorder (RBD), of which constipation and RBD emerge earlier than other prodromes (as early as 15 years before the onset of motor function issues)^1^. Among these prodromal symptoms, RBD has a strong association with the risk of LBD. Several studies have reported the validity of questionnaires for detecting probable RBD, although the accuracy of them is less than polysomnography^2–5^.

Early identification of individuals at risk for LBD is important, as motor and cognitive defects in LBD are progressive after diagnosis, and more than 50% of dopaminergic neurons within the substantia nigra are degenerated by the time of the clinical diagnosis of PD^6^. Various studies have thus been initiated to identify individuals at risk for LBD. Most studies have recruited elderly subjects with prodromal features, gene mutations, or a relevant family history, focusing on olfaction and RBD as prodromes. In these studies, longitudinal follow-up of the motor and cognitive functions, non-motor symptoms (e.g. hyposmia and RBD), blood and spinal fluid biomarkers, transcranial ultrasound, and dopamine transporter (DaT) single-photon-emission computed tomography (SPECT) were used to track the incidence of PD or DLB^7–10^.

DaT-SPECT and myocardial metaiodobenzylguanidine (MIBG) scintigraphy are imaging diagnostic tools for LBD. DaT-SPECT detects degeneration of dopaminergic neurons within the substantia nigra with LBD even before the onset of motor symptoms, but defects in DaT-SPECT are not specific to LBD. In contrast, myocardial MIBG scintigraphy, which visualizes cardiac sympathetic denervation, is a sensitive, specific imaging marker for LBD, even at an early stage^11–13^. The uptake of MIBG is associated with the olfactory function in patients with PD^14^. Previous studies have shown that a decreased cardiac uptake of MIBG frequently appears in patients with idiopathic RBD, suggesting that this modality is a useful diagnostic tool for prodromal-stage LBD^15^. However, few studies have conducted myocardial MIBG scintigraphy together with DaT-SPECT in subjects at risk of developing LBD, particularly those without RBD but with other prodromal symptoms.

In our previous study using questionnaires on prodromal symptoms, we found that 5.7% of health checkup examinees with age ≥50 years old had ≥2 prodromal symptoms (dysautonomia, hyposmia, and probable RBD), and defined them as high-risk^16^. Here we analyzed neurological and imaging indices, including DaT-SPECT and MIBG scintigraphy, in the high-risk individuals with multiple prodromal symptoms as well as in low-risk individuals without prodromal symptoms, to clarify the manifest LBD-related changes at possible prodromal subjects and to verify the plausibility of our screening methods using questionnaires.

## Results

### Participant characteristics

**Fig. 1:**
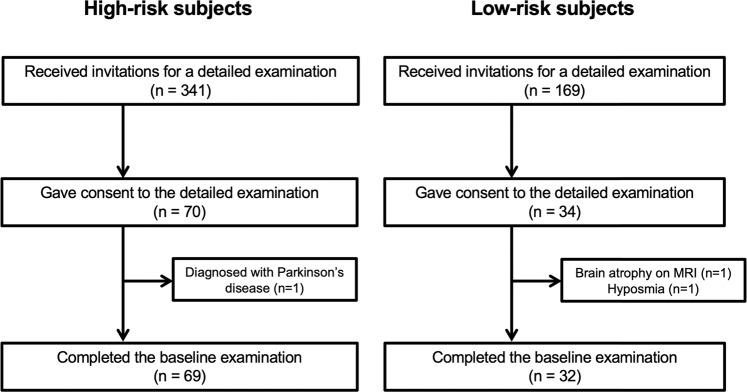
Participant flowchart of this study. We screened health checkup examinees to identify potential participants using the following questionnaires: SCOPA-AUT the Japanese version of the Scale for Outcomes in Parkinson’s disease for Autonomic Symptoms, SAOQ Self-administered Odor Question, RBDSQ REM behavior disorder screening scale, BDI-II Beck Depression Inventory-Second Edition, ESS Epworth Sleepiness Scale, and PASE Physical Activity Scale for the Elderly. We classified subjects who were ≥50 years old and had ≥2 abnormal scores in the SCOPA-AUT, SAOQ, and RBDSQ into the high-risk group. The cut-off value for identifying the high-risk group was 10 for SCOPA-AUT, 90.0% for SAOQ, and 5 for RBDSQ. Subjects who were ≥50 years old and had no abnormalities in all the questionnaires were classified into the low-risk group.

Among the high-risk and low-risk subjects who received an invitation to the present study, 70 and 34, respectively, provided their written informed consent. After exclusion of one high-risk subject who was diagnosed with PD, one low-risk subject with brain atrophy on MRI, and one low-risk subject with subjective hyposmia according to the SAOQ, the baseline cohort included 69 high-risk and 32 healthy subjects (Fig. 1). There were no significant differences between the groups in the age, sex, family history of LBD, smoking history, alcohol consumption rate, caffeine intake, milk intake, dairy product intake, pesticide exposure, and organic solvent exposure. The high-risk subjects, who were selected based on the scores of SCOPA-AUT, SAOQ, and RBDSQ, also had worse scores on BDI-II, ESS, PDQ-39, and QUIP than the low-risk group (Table 1).

**Table 1:** Background characteristics of the participants.

	High-risk	Low-risk	*p*-value^a^
Number (M:F)	69 (42:27)	32 (22:10)	
Age, years	65.2 ± 7.2	63.1 ± 5.0	0.145^b^
Family history of LBD, *n* (%)	9 (13.0)	1 (3.1)	0.163^c^
Smoker, *n* (%)	5 (7.2)	2 (6.3)	1.000^c^
Ex-smoker, *n* (%)	28 (40.6)	10 (31.3)	0.389^c^
Never smoker, *n* (%)	36 (52.2)	20 (62.5)	0.393^c^
Alcohol, *n* (%)	35 (50.7)	21 (65.6)	0.199^c^
Caffeine, mg/day	252.4 ± 170.3	244.9 ± 182.6	0.843^b^
Milk, *n* (%)	37 (53.6)	16 (65.6)	0.831^c^
Dairy products, *n* (%)	48 (69.6)	17 (53.1)	0.123^c^
Pesticides, *n* (%)	6 (8.7)	1 (3.1)	0.427^c^
Organic solvent, *n* (%)	3 (4.3)	2 (6.3)	0.651^c^
PASE	152.9 ± 113.9	106.0 ± 62.9	0.038^d^
SCOPA-AUT	10.5 ± 5.0	2.1 ± 1.7	<0.001^d^
SCOPA-AUT abnormal, %	35 (50.7)	0 (0.0)	<0.001^c^
SAOQ, %	83.2 ± 24.7	99.8 ± 1.1	<0.001^d^
SAOQ abnormal, %	29 (42.0)	0 (0.0)	<0.001^c^
RBDSQ	4.3 ± 2.8	0.7 ± 0.8	<0.001^d^
RBDSQ abnormal, %	27 (39.1)	0 (0.0)	<0.001^c^
BDI-II	11.1 ± 7.3	2.0 ± 2.1	<0.001^d^
ESS	9.2 ± 5.1	4.7 ± 2.8	<0.001^d^
PDQ-39	11.7 ± 9.1	1.2 ± 1.5	<0.001^d^
QUIP	1.0 ± 1.5	0.1 ± 0.4	<0.001^d^

*PASE* Physical Activity Scale for the Elderly, *SCOPA-AUT* the Japanese version of the Scale for Outcomes in Parkinson’s disease for Autonomic Symptoms, *SAOQ* Self-administered Odor Question, *RBDS* RBD screening scale, *BDI-II* Beck Depression Inventory-Second Edition, *ESS* Epworth Sleepiness Scale, *PDQ-39* Parkinson’s Disease Questionnaire-39, *QUIP* Questionnaire for Impulsive-Compulsive Disorders in Parkinson’s Disease.

^a^Comparison between the high-risk group and the low-risk group.

^b^*p*-values were determined by Student’s *t*-test.

^c^*p*-values were determined by Fisher’s exact test.

^d^*p*-values were determined by Mann–Whitney *U*-test.

Data represent the mean ± standard deviation or value (%).

### Manifest LBD-related features

**Fig. 2:**
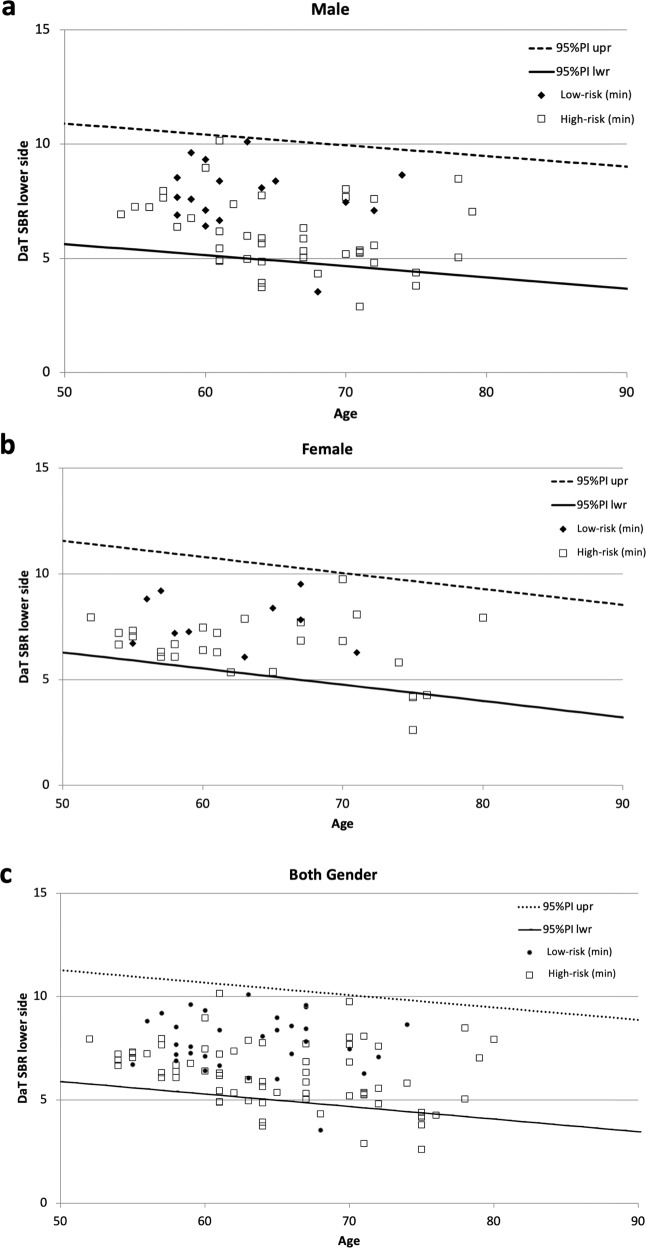
Scatterplot of SBR by age for the high-risk and low-risk subjects. SBR values of DaT-SPECT of the high-risk and low-risk subjects were plotted with lines showing the upper and lower limits of the 95% confidence interval in healthy subjects^27^. **a** males; **b** females; **c** both genders. DaT dopamine transporter, SBR specific binding ratio.

The high-risk subjects had significantly worse scores of the Movement Disorder Society-Sponsored Revision of the Unified Parkinson’s Disease Rating Scale MDS-UPDRS, Stroop test, line orientation test, and OSIT-J scores than the low-risk group (Table 2). The high-risk subjects also had significantly lower SBR values of DaT-SPECT and significantly higher proportions of individuals with DaT abnormalities than the low-risk group (Table 2, Fig. 2). About one-third of the high-risk subjects showed abnormalities in either or both DaT-SPECT or MIBG scintigraphy (Table 2). The high-risk subjects with all three prodromal symptoms (dysautonomia, hyposmia, and probable RBD) had significantly worse scores of the MDS-UPDRS part 1 and 3, OSIT-J, SAOQ, and RBDSQ than those with two symptoms (Supplementary Table 1).

**Table 2:** Lewy body disease-related neurological and imaging indices of the participants.

	High-risk	Low-risk	*p*-value^a^
Number (M:F)	69 (42:27)	32 (22:10)	
Age, years	65.2 ± 7.2	63.1 ± 5.0	0.145^b^
MDS-UPDRS total	14.2 ± 7.9	3.4 ± 2.7	<0.001^b^
MDS-UPDRS part 1	7.7 ± 3.6	1.5 ± 1.6	<0.001^b^
MDS-UPDRS part 2	2.2 ± 2.8	0.3 ± 1.2	0.002^b^
MDS-UPDRS part 3	4.2 ± 3.6	1.6 ± 1.5	<0.001^b^
MDS-UPDRS part 4	0.0 ± 0.0	0.0 ± 0.0	
MoCA-J	27.2 ± 1.9	27.6 ± 2.3	0.335^b^
Stroop test part 2 – part 1, sec	13.9 ± 8.4	9.5 ± 4.7	0.007^b^
Trail making test part B – part A, sec	47.7 ± 22.6	43.2 ± 26.7	0.338^b^
Line orientation test	16.9 ± 2.6	18.5 ± 2.2	0.006^b^
Pareidolia test	0.12 ± 0.40	0.03 ± 0.18	0.259^b^
OSIT-J	8.7 ± 3.1	10.5 ± 1.5	0.002^b^
CVRR rest, %	3.36 ± 1.46	3.08 ± 1.65	0.420^b^
CVRR deep breath, %	5.14 ± 2.81	5.02 ± 2.46	0.837^b^
DaT-SPECT SBR lower	6.24 ± 1.53	7.79 ± 1.37	<0.001^b^
DaT-SPECT Z-score lower	−1.03 ± 1.07	0.08 ± 1.03	<0.001^b^
DaT-SPECT SBR higher	6.58 ± 1.52	8.06 ± 1.29	<0.001^b^
DaT-SPECT Z-score higher	−0.79 ± 1.07	0.29 ± 0.98	<0.001^b^
DaT-SPECT Asymmetry Index, %	5.7 ± 4.6	3.9 ± 6.0	<0.112^b^
DaT abnormal, %	17 (24.6)	2 (6.3)	0.030^c^
MIBG H/M early	2.89 ± 0.64	2.97 ± 0.45	0.496^b^
MIBG H/M delay	3.10 ± 0.91	3.28 ± 0.65	0.309^b^
MIBG washout ratio, %	21.0 ± 20.7	16.3 ± 14.3	0.251^b^
MIBG abnormal, %	13 (18.9)	2 (6.3)	0.135^c^
DaT and/or MIBG abnormal	23 (33.3)	2 (6.3)	0.003^c^

*MDS-UPDRS* Movement Disorder Society-sponsored revision of the Unified Parkinson’s Disease Rating Scale, *MoCA-J* the Japanese version of the Montreal Cognitive Assessment, *OSIT-J* the odor stick identification test for Japanese, *CVRR* coefficient of variation of RR intervals, *DaT* dopamine transporter, *MIBG* metaiodobenzylguanidine.

^a^Comparison between the high-risk group and the low-risk group.

^b^*p*-values were determined by Student’s *t*-test.

^c^*p*-values were determined by Fisher’s exact test.

Data represnt the mean±standard deviation or value (%).

### Differences in clinical features of high-risk subjects with and without imaging abnormalities

To investigate the relationship between the neurological function and imaging features in high-risk subjects, we classified them into the following subgroups: subjects with abnormalities in both DaT and MIBG, those with abnormalities only in DaT, those with abnormalities only in MIBG, and those with normal DaT and MIBG findings.

The high-risk subjects with both DaT and MIBG abnormalities were older with significantly worse MDS-UPDRS part 3, Stroop test, trail making test, line orientation test, and OSIT-J scores than the high-risk subjects without imaging abnormalities (Table 3). Even after adjusting for age with an ANCOVA, the high-risk subjects with DaT and MIBG abnormalities had significantly worse MDS-UDPRS part 3 and OSIT-J scores than the high-risk subjects with no imaging abnormalities (Supplementary Table 2). The high-risk subjects with abnormal DaT and normal MIBG findings had significantly higher MDS-UPDRS part 3 scores than the high-risk subjects with no imaging abnormalities, and the high-risk subjects with normal DaT and abnormal MIBG findings had significantly worse OSIT-J and RBDSQ scores than the high-risk subjects with no imaging abnormalities.

**Table 3:** Clinical indices of the high-risk subjects with and without imaging defects.

	DaT & MIBG abnormal (B)	DaT abnormal (D)	MIBG abnormal (M)	DaT & MIBG Normal (N)	*p*-values		
					B vs. N	D vs. N	M vs. N
Number (M:F)	7 (4:3)	10 (8:2)	6 (5:1)	46 (25:21)			
Age, years	74.1 ± 2.6	66.3 ± 5.1	65.5 ± 3.6	63.5 ± 7.4	<0.001^a^	0.515	0.851
MDS-UPDRS total	21.4 ± 12.4	16.4 ± 5.4	12.3 ± 5.1	12.8 ± 7.3	0.018^a^	0.413	0.999
MDS-UPDRS part 1	8.0 ± 4.3	7.9 ± 1.6	7.8 ± 2.7	7.7 ± 4.0	0.989^a^	0.993	0.998
MDS-UPDRS part 2	3.7 ± 4.0	2.6 ± 2.7	1.7 ± 1.5	2.0 ± 2.8	0.319^a^	0.863	0.996
MDS-UPDRS part 3	9.7 ± 4.8	5.9 ± 3.0	2.8 ± 2.2	3.2 ± 2.8	<0.001^a^	0.041	0.988
MDS-UPDRS part 4	0.0 ± 0.0	0.0 ± 0.0	0.0 ± 0.0	0.0 ± 0.0			
MoCA-J	26.1 ± 3.0	26.7 ± 2.5	27.7 ± 1.5	27.4 ± 1.6	0.298^a^	0.667	0.976
Stroop test part 2 – part 1, sec	22.0 ± 17.5	17.4 ± 7.2	8.7 ± 3.5	12.6 ± 6.0	0.013^a^	0.218	0.576
Trail making test part B – part A, sec	66.5 ± 26.7	58.3 ± 24.4	32.0 ± 16.0	44.6 ± 20.1	0.038^a^	0.186	0.431
Line orientation test	14.9 ± 3.1	16.0 ± 2.8	17.7 ± 2.1	17.4 ± 2.4	0.048^a^	0.315	0.992
Pareidolia test	0.29 ± 0.76	0.20 ± 0.42	0.00 ± 0.00	0.09 ± 0.35	0.537^a^	0.806	0.944
OSIT-J	4.9 ± 3.6	8.1 ± 3.2	6.3 ± 2.9	9.7 ± 2.4	<0.001^a^	0.269	0.019
CVRR rest, %	2.92 ± 2.69	2.62 ± 1.91	3.06 ± 0.87	3.22 ± 1.50	0.961^a^	0.662	0.995
CVRR deep breath, %	4.48 ± 4.66	4.50 ± 2.18	7.99 ± 1.96	5.01 ± 2.53	0.949^a^	0.930	0.041
DaT-SPECT SBR lower	4.02 ± 1.00	4.65 ± 0.58	6.41 ± 1.21	6.91 ± 1.17	<0.001^a^	<0.001	0.646
DaT-SPECT Z-score lower	−2.27 ± 0.76	−2.12 ± 0.41	−0.87 ± 0.76	−0.64 ± 0.95	<0.001^a^	<0.001	0.901
DaT-SPECT SBR higher	4.42 ± 1.03	5.03 ± 0.57	6.63 ± 1.19	7.24 ± 1.18	<0.001^a^	<0.001	0.483
DaT-SPECT Z-score higher	−1.97 ± 0.79	−1.84 ± 0.40	−0.71 ± 0.76	−0.39 ± 0.96	<0.001^a^	<0.001	0.784
DaT-SPECT Asymmetry Index, %	9.7 ± 7.8	7.9 ± 5.9	3.5 ± 1.6	4.9 ± 3.5	0.024^a^	0.138	0.853
MIBG H/M early	1.74 ± 0.32	3.32 ± 0.36	1.97 ± 0.24	3.09 ± 0.41	<0.001^a^	0.244	<0.001
MIBG H/M delay	1.40 ± 0.25	3.62 ± 0.57	1.67 ± 0.30	3.43 ± 0.53	<0.001^a^	0.623	<0.001
MIBG washout ratio, %	62.7 ± 8.0	12.9 ± 9.5	50.5 ± 12.5	12.9 ± 11.2	<0.001^a^	0.999	<0.001
PASE	106.9 ± 60.7	177.4 ± 76.2	111.6 ± 69.7	160.0 ± 130.5	0.587^b^	0.961	0.701
SCOPA-AUT	11.3 ± 6.2	11.7 ± 5.4	9.0 ± 2.9	10.3 ± 5.1	0.955^b^	0.826	0.906
SCOPA-AUT abnormal, %	3 (42.9)	7 (70.0)	2 (33.3)	23 (50.0)	1.000^c^	1.000	1.000
SAOQ, %	53.2 ± 39.7	84.2 ± 23.0	63.0 ± 37.3	90.7 ± 13.2	<0.001^b^	0.769	0.013
SAOQ abnormal, %	5 (71.4)	3 (30.0)	4 (66.7)	17 (37.0)	0.680^c^	1.000	1.000
RBDSQ	3.9 ± 2.7	3.5 ± 3.0	7.2 ± 2.8	4.2 ± 2.7	0.990^b^	0.869	0.042
RBDSQ abnormal, %	2 (28.6)	3 (30.0)	5 (83.3)	17 (37.0)	1.000^c^	1.000	0.430
BDI-II	9.1 ± 6.2	10.7 ± 5.3	10.7 ± 5.6	11.6 ± 8.2	0.810^b^	0.983	0.990
ESS	6.3 ± 4.5	9.2 ± 4.7	9.5 ± 4.7	9.5 ± 5.3	0.331^b^	0.998	1.000
PDQ-39	8.3 ± 6.4	10.0 ± 5.9	10.0 ± 5.6	13.0 ± 10.3	0.500^b^	0.719	0.837
QUIP	0.6 ± 1.5	1.3 ± 2.5	0.2 ± 0.4	1.1 ± 1.3	0.780^b^	0.969	0.411

*MDS-UPDRS* Movement Disorder Society-sponsored revision of the Unified Parkinson’s Disease Rating Scale, *MoCA-J* the Japanese version of the Montreal Cognitive Assessment, *OSIT-J* the odor stick identification test for Japanese, *CVRR* coefficient of variation of RR intervals, *DaT* dopamine transporter, *MIBG* metaiodobenzylguanidine, *PASE* Physical Activity Scale for the Elderly, *SCOPA-AUT* the Japanese version of the Scale for Outcomes in Parkinson’s disease for Autonomic Symptoms, *SAOQ* Self-administered Odor Question, *RBDSQ* RBD screening scale, *BDI-II* Beck Depression Inventory-Second Edition, *ESS* Epworth Sleepiness Scale, *PDQ-39* Parkinson’s Disease Questionnaire-39, *QUIP* Questionnaire for Impulsive-Compulsive Disorders in Parkinson’s Disease.

^a^*p*-values were determined by a one-way ANOVA with Dunnett post-hoc test.

^b^*p*-values were determined by Kruskal–Wallis test with Steel post-hoc test.

^c^*p*-values were determined by Fisher’s exact test adjusted by Bonferroni correction.

Data represent the mean±standard deviation or value (%).

We next investigated the manifest LBD-related features of high-risk individuals with normal imaging findings. The high-risk subjects without DaT or MIBG abnormalities had significantly higher MDS-UPDRS scores and worse Stroop test and line orientation test scores than the low-risk group (Supplementary Table 3). They also had significantly lower DaT-SPECT SBR values than the low-risk group, although the values were within the normal range. There was no significant difference in the H/M ratio of MIBG between the two groups. The scores of motor and non-motor symptoms, including ones not used for identifying high-risk subjects, were significantly worse in the high-risk group with no imaging abnormalities than in the low-risk group.

### Correlation between imaging findings and clinical scores in high-risk subjects

**Fig. 3:**
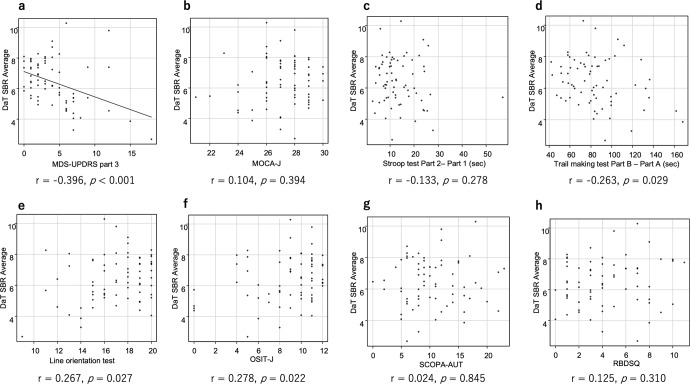
The correlation between DaT-SPECT SBR and clinical indices in the high-risk subjects. Pearson’s correlation test comparing the DaT SBR and motor (**a**) and cognitive (**b**–**e**) functions as well as non-motor symptoms (**f**–**h**) in the participants. DaT dopamine transporter, SBR specific binding ratio, MDS-UPDRS Movement Disorder Society-sponsored revision of the Unified Parkinson’s Disease Rating Scale, MoCA-J the Japanese version of the Montreal Cognitive Assessment, OSIT-J the odor stick identification test for Japanese, SCOPA-AUT the Japanese version of the Scale for Outcomes in Parkinson’s disease for Autonomic Symptoms, RBDSQ RBD screening scale.

**Fig. 4:**
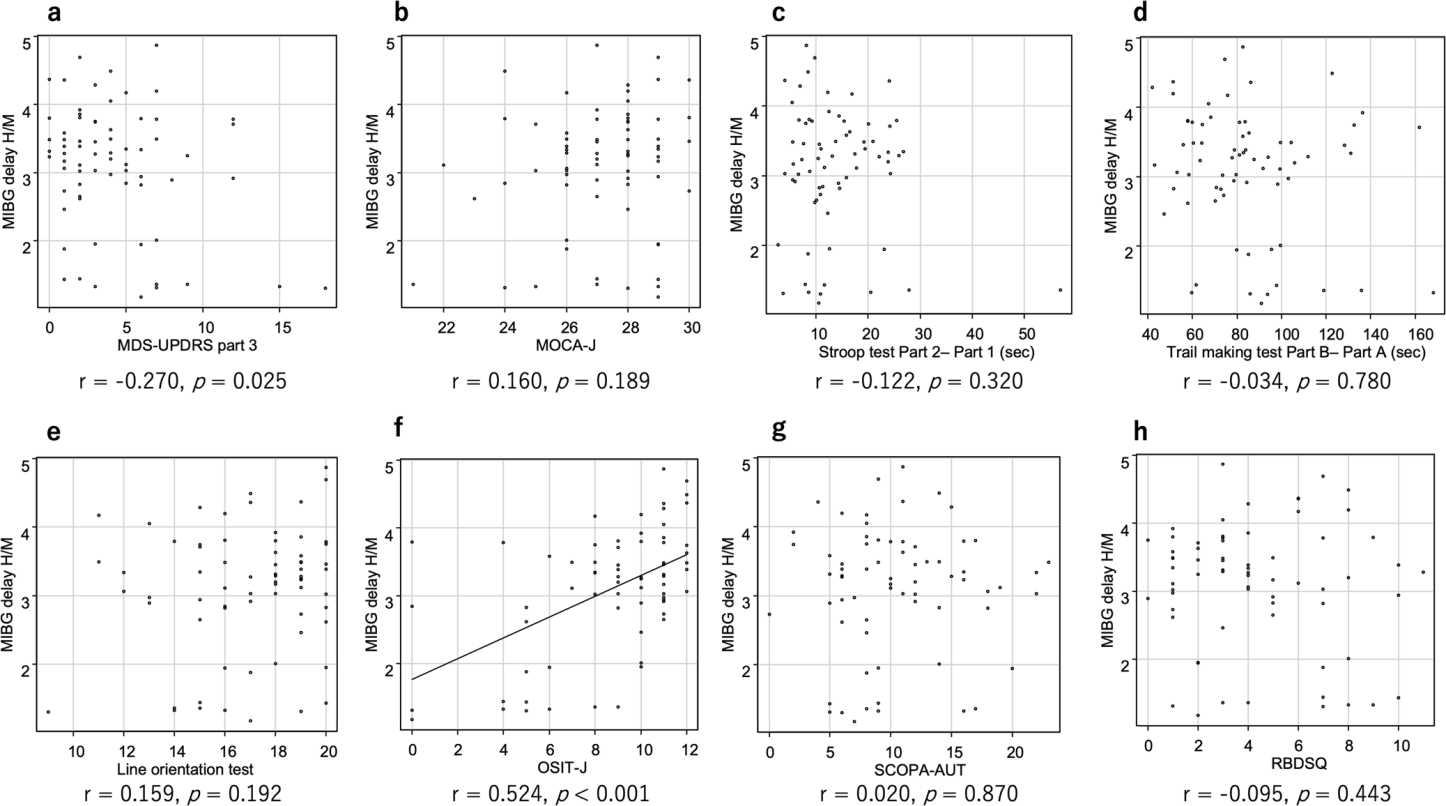
The correlation between delayed MIBG H/M and clinical indices in the high-risk subjects. Pearson’s correlation test comparing the delayed MIBG H/M ratio and motor (**a**) and cognitive (**b**–**e**) functions as well as non-motor symptoms (**f**–**h**) in the participants. *MIBG* metaiodobenzylguanidine, *MDS-UPDRS* Movement Disorder Society-sponsored revision of the Unified Parkinson’s Disease Rating Scale, *MoCA-J* the Japanese version of the Montreal Cognitive Assessment, *OSIT-J* the odor stick identification test for Japanese, *SCOPA-AUT* the Japanese version of the Scale for Outcomes in Parkinson’s disease for Autonomic Symptoms; *RBDSQ* RBD screening scale.

An analysis of the correlation between DaT-SPECT SBR and each clinical score in the high-risk subjects showed a mild negative correlation between DaT-SPECT SBR and MDS-UPDRS part 3 (Fig. 3). By contrast, an analysis of the correlation between the delayed MIBG H/M ratio and each clinical score in high-risk subjects showed a moderate positive correlation between the delayed MIBG H/M ratio and the OSIT-J score (Fig. 4).

### Anthropometric and blood test results of high-risk subjects

The female subjects in the high-risk group had significantly lower hematocrit levels than the low-risk group, but there were no significant differences in the other parameters (Supplementary Table 4). The male high-risk subjects also tended to have lower hematocrit levels, although the difference from the low-risk group was not statistically significant.

## Discussion

The present study showed that high-risk subjects identified by a questionnaire survey on prodromal symptoms (dysautonomia, hyposmia, and probable RBD) of health checkup examinees had mild changes in motor and cognitive scales with a higher prevalence of imaging abnormalities on DaT-SPECT than normal subjects without prodromal symptoms. In addition, those high-risk subjects also had worse scores on other questionnaires about depression and daytime sleepiness, indicating that they had multiple non-motor symptoms similar to patients with LBD. The high rate of LBD-related neurological and imaging defects in the high-risk subjects confirms the utility of a questionnaire on prodromal symptoms as a method of screening individuals at high risk of developing LBD.

Regarding the relationship between functional and imaging features, we found that DaT-SPECT abnormalities were associated with motor impairment, while MIBG scintigraphy abnormalities were associated with olfactory impairment. These findings were in line with the two major pathological development patterns of PD (brain-first and body-first) proposed based on chronological changes in imaging biomarkers^17^. In the present study, the presence of DaT-SPECT abnormalities and absence of MIBG abnormalities seemed to correspond to brain-first PD, wherein patients develop dopaminergic degeneration prior to myocardial sympathetic denervation. In contrast, a decreased uptake of MIBG without DaT abnormalities was likely indicative of body-first PD, wherein the disease is initiated by non-motor features, such as myocardial sympathetic denervation, olfactory dysfunction, or RBD^18^. In addition, the high-risk group with both DaT and MIBG defects were characterized by an old age and motor, cognitive, and olfactory impairment, suggesting that LBD-related pathological changes developed more extensively in such subjects and possibly closer to the onset of LBD than in other groups. Taken together, our results appear to support the different patterns of pathological development during a prodromal phase of LBD.

In the present study, the high-risk subjects with normal DaT and MIBG findings still had more minor motor and cognitive impairment and milder reductions in DaT-SPECT SBR values, albeit still within the normal range, than low-risk subjects. This result suggests that the presence of prodromal symptoms without overt imaging defects may be associated with early pathological changes of LBD. Given that these high-risk subjects’ ages were about three years younger than the groups with defects in either DaT-SPECT or MIBG scintigraphy, a longitudinal study is needed to determine if imaging abnormalities of DaT or MIBG appear within the next few years in high-risk subjects with normal imaging. We are currently performing a prospective longitudinal study, the results of which are expected to provide insight into the progression of LBD at a very early stage.

In our study, both the high-risk and low-risk subjects were identified through a questionnaire survey linked to a health checkup. This enabled us to investigate the changes in anthropometric and blood markers in the high-risk subjects of our cohort. Our results demonstrated that female high-risk subjects had decreased hematocrit with normal hemoglobin values compared with female subjects without prodromal symptoms, a pattern indicating a mild iron defect. Although not significant, similar findings were shown for the male high-risk subjects in the present study. These results were in line with literature indicating iron deficiency anemia as associated with a risk of PD onset^19^ but were not identical to our previous finding that male (but not female) subjects at risk of LBD had decreased hematocrit values^16^. The discrepancy between the previous and present studies may stem from the size and age of the population examined, but further studies will be necessary to clarify the systemic changes in the prodromal stage of LBD.

The present study was associated with several limitations. First, this study had a small sample size of high-risk and low-risk subjects, and the data were limited to Asians. We thus cannot exclude the possible selection bias. In particular, there were fewer participants in the low-risk group, partly because participation in the present study was voluntary and the low-risk subjects were less motivated to participate. We therefore plan to increase the number of institutes in our cohort study to expand the sample size. Second, in the present study, RBD was assessed only with the RBDSQ questionnaire and not with polysomnography. Although a previous study showed that RBD can be diagnosed with an RBDSQ cut-off value of 5, as was used in our study, with a sensitivity of 88.5% and specificity of 96.9% in Japanese subjects^20^, it is necessary to confirm the findings of REM sleep without atonia on polysomnography to make a definitive diagnosis of RBD. Third, only the baseline, but not followed-up, findings were available in the present study. It is thus important to determine the natural history of neurological and imaging indices as well as the incidence of LBD conversion in these high-risk individuals through further longitudinal analyses and pathological confirmation.

In summary, our study demonstrates that high-risk subjects for LBD identified in a questionnaire survey on prodromal symptoms had low SBR of DaT-SPECT. A decreased uptake in DaT-SPECT was associated with motor impairment, and a reduced MIBG uptake was associated with olfactory dysfunction. A longitudinal evaluation of both the high-risk and low-risk groups is warranted to determine the natural history of prodromal LBD.

## Methods

### Ethics approval

This study was conducted in accordance with the 1964 Declaration of Helsinki and its later amendments, the Ethics Guidelines for Human Genome/Gene Analysis Research, and the Ethical Guidelines for Medical and Health Research Involving Human Subjects endorsed by the Japanese government. The study protocol was approved by the Ethics Review Committee of Nagoya University Graduate School of Medicine (No. 2017-0521). All participants provided their written informed consent before participation in the study.

### Participants

The Nagoya-Takayama preclinical/prodromal Lewy body disease study (NaT-PROBE study) is a prospective, longitudinal, multi-center, community-based cohort study coordinated by Nagoya University School of Medicine^16^. Since March 2017, we have been conducting a survey of prodromal symptoms in healthy individuals who visited Kumiai Kosei Hospital or Daido Clinic, Japan, for their annual health checkup. In Japan, regular medical checkups, which are obligatory for employees, are performed annually according to the Industrial Safety and Health Law. We used health checkup cohorts in Kumiai Kosei Hospital and Daido Clinic to screen for prodromal symptoms in the Japanese community-based population by using the following questionnaires: the Japanese version of the Scale for Outcomes in Parkinson’s disease for Autonomic Symptoms (SCOPA-AUT); the Self-administered Odor Question (SAOQ); the RBD screening scale (RBDSQ); the Beck Depression Inventory-Second Edition (BDI-II); the Epworth Sleepiness Scale (ESS); and the Physical Activity Scale for the Elderly (PASE). Based on the results of our previous study^16^, we classified subjects who were ≥50 years old and had ≥2 abnormal scores in the SCOPA-AUT, SAOQ, and RBDSQ into the high-risk group. The cut-off value for identifying the high-risk group was 10 for SCOPA-AUT, 90.0% for SAOQ, and 5 for RBDSQ^16^. Subjects who were ≥50 years old and had no abnormalities in any of the questionnaires were classified into the low-risk group.

Between April 2018 and March 2021, we sent invitations to undergo neurological and imaging examinations for LBD to 341 high-risk and 169 low-risk individuals who were identified in the NaT-PROBE study. On the high-risk and low-risk subjects who gave written consent for the present study, we performed a detailed examination of the motor, cognitive, and physiological functions, non-motor symptoms, and DaT-SPECT and cardiac MIBG scintigraphy. All the examinations for both low- and high-risk groups were performed for solely research purposes with the funds supporting this study.

We excluded participants who had PD or DLB at the baseline assessment. In addition, participants with a history of psychiatric or neurological disorders other than depression and those with brain MRI abnormalities were also excluded from this study. Serum, plasma, and urine samples were collected from all participants for future research use.

### Motor and cognitive examinations

The Movement Disorder Society-sponsored revision of the Unified Parkinson’s Disease Rating Scale (MDS-UPDRS) was scored by neurologists who were certified as MDS-UPDRS evaluators (M.H., Y.S., K.H. and K.Y.) for assessing motor and non-motor symptoms related to PD. The Japanese version of the Montreal Cognitive Assessment (MoCA-J) was administered to assess the general cognitive function, the Stroop test and trail making test were conducted to assess the frontal lobe function, and the line orientation test and pareidolia test were conducted to assess the visuospatial cognitive function.

### Physiological function tests

We conducted the Odor Stick Identification Test for Japanese (OSIT-J) to assess objective olfactory dysfunction. The OSIT-J is a stick-type olfaction test, consisting of 12 odorants typically familiar to Japanese subjects, and is a simple test that takes approximately 10 min to complete^21^. The CVRR was measured as previously described^22^. In brief, participants maintained a supine position with normal breathing for more than 5 min. The resting-CVRR was calculated as a percentage of the standard deviation of the last 100 R-R intervals divided by their mean. To assess the deep breath-CVRR, participants took 12 deep breaths over a 2-min period, and the deep breath-CVRR was calculated using the first 100 successive electrocardiogram R-R intervals.

### Imaging tests

We conducted DaT-SPECT imaging with [123I]FP-CIT to detect presynaptic dopamine neuronal dysfunction. All patients were scanned at Nagoya University Hospital or Kumiai Kosei Hospital. DaT-SPECT data were acquired using a Symbia T (Siemens, Erlangen, Germany), equipped with a low-medium energy general-purpose (LMEGP) collimator at Nagoya University Hospital, and an Infinia (GE Healthcare, Milwaukee, WI, USA), equipped with a low-energy high-resolution (LEHR) collimator at Kumiai Kosei Hospital. Ninety projections over 360° orbit with 2 detectors were acquired on a 128 × 128 matrix (zoom factor, 1.45), giving a pixel size of 3.3 mm and acquisition time of 28 min. The main energy window was 159 keV±10%, and 2 subwindows were set at 8% at both ends of the main window. Images were reconstructed using a three-dimensional ordered subset expectation maximization method (3D-OSEM) (iteration, 6; subset, 8) and Gaussian filter full width at half maximum (FWHM) 6 mm with attenuation correction (AC) by computed tomography (CT) and scatter correction (SC) using the triple energy window method^23^ in the same way as in a recent Japanese study^24^ on DaT-SPECT. The specific binding ratio (SBR) values were obtained for both the right and left striatum regions. The Southampton method is widely used in Japan and uses a large volume of interest (VOI), including the entire striatum, to correct the partial-volume effect (PVE)^25^. The SPECT count density computed by this method was defined as CSouthampton. We used the software program DaTView (AZE, Tokyo, Japan), which adopted the Southampton method to compute the CSouthampton for the left and right striatum and BG regions. We defined the most affected striatum side as the ‘lower side’ and the opposite side as the ‘higher side’ with SBR values. We used the average of the right and left sides as the SBR ‘average’. The asymmetry index of SBR was calculated using the following equation: asymmetry index [%] = (SBR_predominantly affected_ – SBR_less affected_) × 2/(SBR_predominantly affected_ + SBR_less affected_) × 100^25,26^. The decrease in SBR was evaluated in reference to the values of Japanese volunteers^27^.

Cardiac [^123^I]MIBG scintigraphy (123I-MIBG) was performed to assess postganglionic cardiac autonomic denervation. MIBG (111 mBq) was injected intravenously into the participants. Early images were obtained 15 min after the injection, and delayed images were obtained after 4 h. The myocardial MIBG uptake was measured using the heart-to-mediastinum ratio (H/M ratio) according to methods described previously^22^. None of our patients had taken drugs known to affect the MIBG uptake (e.g. tricyclic antidepressants, Ca^2+^ blockers, or selegiline). Normal values for myocardial MIBG scintigraphy are ≥2.2 for the early H/M ratio, ≥2.2 for the delayed H/M ratio, and ≤34% for the washout rate^28^.

### Questionnaires on motor and non-motor symptoms

We used the PASE to evaluate the amount of physical activity, the Japanese version of the SCOPA-AUT to evaluate autonomic dysfunction, the SAOQ to evaluate olfactory dysfunction, the RBDSQ to evaluate RBD, the BDI-II to evaluate depressive symptoms, the ESS to evaluate excessive daytime sleepiness, the Parkinson’s Disease Questionnaire-39 (PDQ-39) to evaluate the PD-specific health-related quality of life, and the Questionnaire for Impulsive-Compulsive Disorders in Parkinson’s Disease (QUIP) to evaluate impulse control disorder.

All scales used in this study were validated for self-administration in a Japanese population^20,29–35^.

### Statistical analyses

All data represent the mean±standard deviation unless otherwise stated. The demographic and clinical scores of the high-risk and low-risk groups were compared using Student’s *t*-test or the Mann–Whitney *U*-test when appropriate. Fisher’s exact test was used to compare categorical variables between groups, and Bonferroni correction was used to calculate adjusted *p*-values for multiple comparisons. For the comparisons among the high-risk subjects with and without abnormal DaT-SPECT and/or MIBG, a parametric one-way analysis of variance (ANOVA) followed by Dunnett tests or non-parametric Kruskal–Wallis tests followed by Steel tests were performed. For the comparison of the high-risk subjects with and without DaT-SPECT and MIBG abnormalities, the analysis was performed after adjusting for age with an analysis of covariance (ANCOVA). Pearson’s correlation coefficient was used to determine the relationships between each clinical score and the DaT-SPECT SBR or delayed MIBG H/M ratio.

*P*-values of <0.05 were considered to indicate statistical significance. Correlation coefficients (*r*) were interpreted as follows: >0.8, very strong; 0.5–0.8, moderately strong; and 0.3–0.5, weak. All statistical analyses were conducted using EZR (Saitama Medical Center, Jichi Medical University, Saitama, Japan)^36^, which is a graphical user interface for R (The R Foundation for Statistical Computing, Vienna, Austria).

### Reporting summary

Further information on research design is available in the Nature Portfolio Reporting Summary linked to this article.

## Supplementary information

Supplemental material

Reporting Summary Flattened

## Data Availability

Data are available on reasonable request. Anonymized data will be shared upon request from a qualified investigator.
