# Supplementary material for: Clinico-imaging features of subjects at risk of Lewy body disease in NaT-PROBE baseline analysis
Source: NPJ Parkinsons Dis. 2023 Apr 26;9:67. doi: 10.1038/s41531-023-00507-y (PMC10133289; doi:10.1038/s41531-023-00507-y)
Supplement: Supplementary file 1 — Supplemental material [file 41531_2023_507_MOESM1_ESM.pdf]

Supplemental data

Clinico-imaging features of subjects at risk of Lewy body disease in NaT-PROBE baseline analysis

Makoto Hattori, MD, PhD, Keita Hiraga, MD, Yuki Satake, MD, Takashi Tsuboi, MD, PhD, Daigo Tamakoshi, MD, Maki Sato, Katsunori Yokoi, MD, PhD, Keisuke Suzuki, MD, PhD, Yutaka Arahata, MD, PhD, Akihiro Hori, MD, PhD, Motoshi Kawashima, MD, Hideaki Shimizu, MD, PhD, Hiroshi Matsuda, MD, PhD, Katsuhiko Kato, MD, PhD, Yukihiko Washimi, MD, PhD, Masahisa Katsuno, MD, PhD

Contents:

Four Supplemental Tables (Supplementary table 1, Supplementary table 2, Supplementary table 3, Supplementary table 4)

Supplementary Table 1. Difference in clinical indices by the number of prodromal symptoms

Number of prodromal symptoms	3	2	*p*-value[a]
Number (M:F)	8 (5:3)	61 (37:24)	
Age, years	65.1±7.2	65.0±7.0	0.947[b]
MDS-UPDRS total	20.5±10.9	13.3±7.1	0.014[b]
MDS-UPDRS part 1	7.7±3.6	1.5±1.6	<0.001[b]
MDS-UPDRS part 2	3.4±4.0	2.0±2.6	0.205[b]
MDS-UPDRS part 3	6.9±5.4	3.9±3.3	0.028[b]
MDS-UPDRS part 4	0.0±0.0	0.0±0.0	
MoCA-J	26.4±1.9	27.3±1.9	0.207[b]
Stroop test part 2 – part 1	13.0±6.9	14.0±8.6	0.744[b]
Trail making test part B – part A	48.6±33.6	47.6±21.1	0.903[b]
Line orientation test	16.9±2.3	17.0±2.7	0.927[b]
Pareidolia test	0.25±0.46	0.10±0.40	0.321[b]
OSIT-J	5.9±3.9	9.0±2.9	0.007[b]
CVRR rest, %	4.05±2.30	2.96±1.52	0.078[b]
CVRR deep breath, %	5.76±1.99	5.06±2.91	0.513[b]
DaT-SPECT SBR lower	6.39±1.96	6.22±1.48	0.779[b]
DaT-SPECT Z-score lower	-0.94±1.37	-1.05±1.04	0.782[b]
DaT-SPECT SBR higher	6.64±1.91	6.57±1.47	0.914[b]
DaT-SPECT Z-score higher	-0.76±1.33	-0.79±1.04	0.923[b]
DaT-SPECT Asymmetry Index, %	4.2±2.4	5.9±4.8	0.345[b]
DaT abnormal, %	2 (25.0)	15 (24.6)	1.000[c]
MIBG H/M early	2.67±0.74	2.92±0.62	0.312[b]
MIBG H/M delay	2.79±1.19	3.14±0.88	0.319[b]
MIBG abnormal, %	3 (37.5)	10 (16.4)	0.167[c]
PASE	124.6±75.5	156.6±118.9	0.463[d]
SCOPA-AUT	12.4±3.1	10.3±5.2	0.128[d]
SAOQ	57.1±26.3	86.3±23.0	0.001[d]
RBDSQ	7.3±1.6	3.9±2.8	0.001[d]

BDI-II	14.8±9.1	10.6±7.1	0.199[d]
ESS	11.9±5.5	8.8±5.0	0.138[d]
PDQ-39	13.6±9.6	11.5±9.1	0.549[d]
QUIP	2.3±2.8	0.8±1.2	0.102[d]

MDS-UPDRS, Movement Disorder Society-sponsored revision of the Unified Parkinson's Disease Rating Scale; MoCA-J, the Japanese version of the Montreal Cognitive Assessment; OSIT-J, the odor stick identification test for Japanese; CVRR, coefficient of variation of RR intervals; DaT, dopamine transporter; MIBG, metaiodobenzylguanidine; PASE, Physical Activity Scale for the Elderly; SCOPA-AUT, the Japanese version of the Scale for Outcomes in Parkinson's disease for Autonomic Symptoms; SAOQ, Self-administered Odor Question; RBDSQ, RBD screening scale; BDI-II, Beck Depression Inventory-Second Edition; ESS, Epworth Sleepiness Scale; PDQ-39, Parkinson's Disease Questionnaire-39; QUIP, Questionnaire for Impulsive-Compulsive Disorders in Parkinson's Disease

[a] Comparison between the triple-risk group and the double-risk group.

[b] *p*-values were determined by Student's *t*-test.

[c] *p*-values were determined by Fisher's exact test.

[d] *p*-values were determined by Mann-Whitney U test.

Data represent the mean±standard deviation or value (%).

Supplementary Table 2. Age-adjusted comparison of clinical indices of the high-risk subjects with DaT and MIBG defects and those without imaging defects

	High-risk with abnormal DaT and MIBG	High-risk with normal DaT and MIBG	*p*-value (ANCOVA)[a]
Number, M:F	7 (4:3)	46 (25:21)	
Age, years	74.1±2.6	63.5±7.4	
MDS-UPDRS total	21.4±12.4	12.8±7.3	0.020
MDS-UPDRS part 1	8.0±4.3	7.7±4.0	0.427
MDS-UPDRS part 2	3.7±4.0	2.0±2.8	0.110
MDS-UPDRS part 3	9.7±4.8	3.2±2.8	<0.001
MDS-UPDRS part 4	0.0±0.0	0.0±0.0	
MoCA-J	26.1±3.0	27.4±1.6	N/A[b]
Stroop test part 2 – part 1, sec	22.0±17.5	12.6±6.0	0.141
Trail making test part B – part A, sec	66.5±26.7	44.6±20.1	0.121
Line orientation test	14.9±3.1	17.4±2.4	0.127
Pareidolia test	0.29±0.76	0.09±0.35	N/A[b]
OSIT-J	4.9±3.6	9.7±2.4	0.001
CVRR rest, %	2.92±2.69	3.22±1.50	0.771
CVRR deep breath, %	4.48±4.66	5.01±2.53	0.950
DaT-SPECT SBR lower	4.02±1.00	6.91±1.17	<0.001
DaT-SPECT Z-score lower	-2.27±0.76	-0.64±0.95	<0.001
DaT-SPECT SBR higher	4.42±1.03	7.24±1.18	<0.001
DaT-SPECT Z-score higher	-1.97±0.79	-0.39±0.96	<0.001
DaT-SPECT Asymmetry Index, %	9.7±7.8	4.9±3.5	0.012
MIBG H/M early	1.74±0.32	3.09±0.41	<0.001
MIBG H/M delay	1.40±0.25	3.43±0.53	<0.001
MIBG washout ratio, %	62.7±8.0	12.9±11.2	<0.001

MDS-UPDRS, Movement Disorder Society-sponsored revision of the Unified Parkinson's Disease Rating Scale; MoCA-J, the Japanese version of the Montreal Cognitive Assessment; OSIT-J, the odor stick identification test for Japanese; CVRR, coefficient of variation of R-R intervals; DaT, dopamine transporter; MIBG, metaiodobenzylguanidine

[a] *p*-values were determined by an age-adjusted analysis of covariance.

[b] A covariance analysis cannot be applied due to interactions.

Data represent the mean±standard deviation.

Supplementary Table 3. Clinical indices of the high-risk subjects with normal imaging findings

	High-risk with normal DaT and MIBG	Low-risk group	*p*-value[a]
Number (M:F)	46 (25:21)	32 (22:10)	
Age, years	63.5±7.4	63.1±5.0	0.798[b]
MDS-UPDRS total	12.8±7.3	3.4±2.7	<0.001[b]
MDS-UPDRS part 1	7.7±4.0	1.5±1.6	<0.001[b]
MDS-UPDRS part 2	2.0±2.8	0.3±1.2	0.002[b]
MDS-UPDRS part 3	3.2±2.8	1.6±1.5	0.004[b]
MDS-UPDRS part 4	0.0±0.0	0.0±0.0	
MoCA-J	27.4±1.6	27.6±2.3	0.608[b]
Stroop test part 2 – part 1, sec	12.6±6.0	9.5±4.7	0.018[b]
Trail making test part B – part A, sec	44.6±20.1	43.2±26.7	0.804[b]
Line orientation test	17.4±2.4	18.5±2.2	0.049[b]
Pareidolia test	0.09±0.35	0.03±0.18	0.415[b]
OSIT-J	9.7±2.4	10.5±1.5	0.091[b]
CVRR rest, %	3.22±1.50	3.08±1.65	0.675[b]
CVRR deep breath, %	5.01±2.53	5.02±2.46	0.980[b]
DaT-SPECT SBR lower	6.91±1.17	7.79±1.37	0.003[b]
DaT-SPECT Z-score lower	-0.64±0.95	0.08±1.03	0.002[b]
DaT-SPECT SBR higher	7.24±1.18	8.06±1.29	0.005[b]
DaT-SPECT Z-score higher	-0.39±0.96	0.29±0.98	0.003[b]
DaT-SPECT SBR average	7.07±1.17	7.93±1.32	0.004[b]
DaT-SPECT Asymmetry Index, %	4.9±3.5	3.9±6.0	0.389[b]
MIBG H/M early	3.09±0.41	2.97±0.45	0.243[b]
MIBG H/M delay	3.43±0.53	3.28±0.65	0.273[b]
MIBG washout ratio, %	12.9±11.2	16.3±14.3	0.192[b]
PASE	160.0±130.5	106.0±62.9	0.034[c]
SCOPA-AUT	10.3±5.1	2.1±1.7	<0.001[c]
SAOQ, %	90.7±13.2	99.8±1.1	<0.001[c]

RBDSQ	4.2±2.7	0.7±0.8	<0.001[c]
BDI-II	11.6±8.2	2.0±2.1	<0.001[c]
ESS	9.5±5.3	4.7±2.8	<0.001[c]
PDQ-39	13.0±10.3	1.2±1.5	<0.001[c]
QUIP	1.1±1.3	0.1±0.4	<0.001[c]

MDS-UPDRS, Movement Disorder Society-sponsored revision of the Unified Parkinson's Disease Rating Scale; MoCA-J, the Japanese version of the Montreal Cognitive Assessment; OSIT-J, the odor stick identification test for Japanese; CVRR, coefficient of variation of RR intervals; MIBG, metaiodobenzylguanidine; PASE, Physical Activity Scale for the Elderly; SCOPA-AUT, the Japanese version of the Scale for Outcomes in Parkinson's disease for Autonomic Symptoms; SAOQ, Self-administered Odor Question; RBDSQ, RBD screening scale; BDI-II, Beck Depression Inventory-Second Edition; ESS, Epworth Sleepiness Scale; PDQ-39, Parkinson's Disease Questionnaire-39; QUIP, Questionnaire for Impulsive-Compulsive Disorders in Parkinson's Disease

[a] Comparison between the high-risk group with normal DaT-SPECT and MIBG findings and the low-risk group.

[b] *p*-values were determined by Student's *t*-test.

[c] *p*-values were determined by Mann-Whitney U test.

Data represent the mean±standard deviation.

Supplementary Table 4. Anthropometric and serological indices in high-risk and low-risk subjects

	Males			Females		
	High-risk group	Low-risk group	*p*-value	High-risk group	Low-risk group	*p*-value
Number	42	22		27	10	
Age, years	65.8±6.5	63.0±4.9	0.109	64.0±8.0	62.1±6.7	0.527
Height, cm	167.3±5.8	169.4±4.5	0.190	156.0±5.1	156.9±4.6	0.662
Weight, kg	65.7±11.8	67.1±8.6	0.667	53.8±7.9	54.3±5.7	0.860
BMI, kg/m^2	23.5±4.0	23.3±2.3	0.892	22.1±3.1	22.1±2.7	0.984
WBC, /μL	5721±1356	6200±1270	0.220	5043±1171	4867±899	0.681
Hemoglobin, g/dL	15.1±1.1	15.1±1.0	0.981	13.7±0.8	14.0±1.0	0.398
RBC, 10^4/μL	480.1±42.1	488.2±29.4	0.478	443.1±28.8	461.6±35.4	0.124
Hematocrit, %	44.0±2.6	45.4±2.4	0.061	40.9±2.2	43.0±2.7	0.025
Platelet, 10^4/μL	21.6±4.3	23.8±4.0	0.073	22.2±5.0	23.5±4.7	0.491
Total protein, g/dL	7.34±0.41	7.22±0.24	0.281	7.25±0.32	7.31±0.34	0.616
Uric acid, mg/dL	5.80±1.10	5.69±1.28	0.750	4.77±1.16	4.99±1.02	0.623
Creatinine, mg/dL	0.88±0.14	0.88±0.10	0.879	0.67±0.15	0.71±0.04	0.505
Glucose, mg/dL	102.3±28.0	99.1±7.5	0.648	95.5±12.8	95.6±6.2	0.984
HbA1c, %	5.89±0.61	5.80±0.25	0.584	5.86±0.34	5.76±0.25	0.396
Triglyceride, mg/dL	125.9±81.4	106.5±44.4	0.360	98.8±59.8	147.7±78.8	0.057
T-Cho, mg/dL	200.0±31.6	208.4±28.6	0.357	224.7±34.2	243.7±42.8	0.182
HDL-Cho, mg/dL	61.8±16.4	59.1±16.3	0.569	71.9±11.7	65.7±15.5	0.214
LDL-Cho, mg/dL	117.7±28.0	121.9±26.1	0.615	130.7±29.8	144.6±34.1	0.251
AST, U/L	25.6±11.0	21.8±5.4	0.180	23.0±6.2	21.8±4.9	0.582
ALT, U/L	25.2±17.8	20.0±11.5	0.270	21.0±7.5	19.1±8.0	0.531
γ-GTP, U/L	42.7±40.3	35.1±20.0	0.460	22.2±12.1	20.7±8.0	0.730
ALP, U/L	213.1±58.5	203.0±31.0	0.504	209.1±45.4	211.2±58.4	0.912
T-bil, mg/dL	0.92±0.43	0.98±0.32	0.624	0.80±0.29	0.92±0.16	0.235

BMI, body mass index; WBC, white blood cells; RBC, red blood cells; HbA1c, hemoglobin A1c; T-Cho, total cholesterol; HDL-Cho, high-density lipoprotein cholesterol; LDL-Cho, low-density lipoprotein cholesterol; AST, aspartate aminotransferase; ALT, alanine aminotransferase; γ-GTP, γ-glutamyl transpeptidase; ALP, alkaline phosphatase; T-bil, total bilirubin.

p-values were determined by Student's t-test.

Data represent the mean±standard deviation.
